# Associations between children’s dietary patterns, excessive weight gain, and obesity risk: cohort study nested to a randomized field trial

**DOI:** 10.1590/1984-0462/2025/43/2024117

**Published:** 2025-03-24

**Authors:** Paola Seffrin Baratto, Caroline Nicola Sangalli, Paula dos Santos Leffa, Julia Luzzi Valmorbida, Marcia Regina Vitolo

**Affiliations:** a Universidade Federal de Ciências da Saúde de Porto Alegre, Porto Alegre, RS, Brazil.

**Keywords:** Cohort studies, Child nutrition, Feeding behavior, Food patterns, Food intake, Pediatric obesity, Estudos de coorte, Nutrição da criança, Comportamento alimentar, Padrões dietéticos, Ingestão de alimentos, Obesidade infantil

## Abstract

**Objective:**

To identify the critical period of excessive weight gain (EWG) in childhood and the dietary patterns associated with it.

**Methods:**

A cohort study nested to a randomized field trial with mother-child pairs interviewed by home visits at ages six months, 12 months, three years, and six years. We selected 715 pregnant women from 20 healthcare centers in southern Brazil. EWG was considered as a change in body mass index-for-age (BMI-for-age) z-score >0.67. Two 24-hour recalls were used to determine the dietary patterns by exploratory factor analysis. The effect of dietary patterns on EWG was assessed by logistic regressions using Generalized Estimating Equations.

**Results:**

The critical period for EWG was from six to 12 months (46.5% BMI variation >0.67; n=228; p≤0.001). Dietary patterns at six months associated with EWG were baby bottles of cow´s milk added to baby cereal flours and added sugar (relative risk – RR 1.43; 95% confidence interval – 95%CI 1.15–1.79; p≤0.001 and RR 1.59; 95%CI 1.28–1.97, p≤0.001); fruit juice and creamy dessert (RR 1.39; 95%CI 1.08–1.33; p≤0.001) and sweet beverages (RR 1.19; 95%CI 1.02–1.38; p=0.024).

**Conclusions:**

The second semester of life was the most critical period for EWG in childhood, influenced mostly by the consumption of cow’s milk added with baby cereal flour and sugar, fruit juice, creamy desserts, and sweet beverages. These findings emphasize the importance of early dietary interventions to promote healthier food choices and prevent EWG during infancy.

## RESUMO

**Objetivo:**

Identificar o período crítico de ganho de peso excessivo (GPE) na infância e os padrões alimentares associados a ele.

**Métodos:**

Estudo de coorte aninhado a ensaio de campo randomizado com pares de mães-filhos entrevistados por meio de visita domiciliar nas idades de seis meses, 12 meses, três anos e seis anos. Foram selecionadas 715 gestantes de 20 centros de saúde do sul do Brasil. O GPE foi considerado como alteração no escore z do índice de massa corporal – IMC para idade >0,67. Dois recordatórios de 24 horas foram utilizados para determinar os padrões alimentares por meio de análise fatorial exploratória. O efeito dos padrões alimentares no GPE foi avaliado por regressões logísticas utilizando Equações de Estimativa Generalizadas.

**Resultados:**

O período crítico para o GPE foi de seis a 12 meses (46,5% variação do IMC >0,67; n=228; p≤0,001). O padrão alimentar aos seis meses associado ao GPE foi composto de mamadeiras com leite de vaca com acréscimo de cereais infantis e açúcar (risco relativo – RR 1,43; intervalo de confiança de 95% – IC95% 1,15–1,79; p≤0,001 e RR 1,59; IC95% 1,28–1,97, p≤0,001); suco de frutas e sobremesa cremosa (RR 1,39; IC95% 1,08–1,33; p≤0,001) e bebidas açucaradas (RR 1,19; IC95% 1,02–1,38; p=0,024).

**Conclusões:**

O período crítico para o GPE ocorreu dos seis aos 12 meses de idade. Os padrões alimentares associados ao GPE durante esse período incluíam leite de vaca acrescentado de cereais infantis e açúcar, bem como suco de frutas, sobremesas cremosas e bebidas açucaradas. Estas descobertas enfatizam a importância de intervenções dietéticas precoces para promover escolhas alimentares mais saudáveis e prevenir o GPE durante a infância.

## INTRODUCTION

The world has experienced a widespread increase in the prevalence of childhood obesity over the past three decades, especially in urban areas of developing countries facing rapid socioeconomic transitions. In 2022, the World Health Organization (WHO) estimated that 37 million children younger than five years were overweight or obese.^1^ In Brazil, a recent national survey showed that 28.3% of children under five years of age were overweight and obese,^2^ and a cohort study in Pelotas showed that excess weight in children aged 12 months increased by 88% between 1982 and 2015.^3^ The high prevalence of obesity in children raises the red flag to the increased risk of comorbidities associated with cardiovascular diseases, such as hypertension, atherosclerosis, type 2 diabetes mellitus, stroke, osteoarthritis, sleep apnea, respiratory problems, and cancer.^4^

The fundamental cause of obesity is an imbalance between energy consumed and expended, which can be affected by a complex interplay of genetic and non-genetic factors.^5^ Environmental and social behaviors may also influence excessive weight gain during pregnancy and early childhood and long-term energy balance programming.^6^ In this scenario, dietary patterns experienced in the first thousand days of life are among the main contributing factors to develop the food preferences that will be reproduced throughout adult life.^7^ The time and quality of complementary feeding could reinforce the innate preference for sweet taste and discourage the consumption of *in-natura* foods.^8^

The influence of dietary patterns on anthropometric outcomes in childhood has been recently studied in many countries, such as South Africa^9^ and United States.^10^ Similar studies conducted so far in Brazil evaluated children from 1–4 years,^11^ 4–7 years,^12^ 7–10 years^13^ or 7–16 years.^14^ The studies with Brazilian data including children under two years of age investigated associated factors and dietary patterns, but not the dietary patterns and anthropometric variables.^15,16,17^ Considering that the first two years of life are the window of opportunity to promote healthy infant feeding practices, we assume that dietary patterns experienced by Brazilian infants in this period should also be investigated as potential determinants of childhood obesity.

Therefore, this study aimed to investigate the association between feeding practices and childhood obesity. The specific objectives of this longitudinal study were, first, to identify the period of rapid weight gain in a cohort from birth to six years across age time points; second, to determine the diet patterns at the age time point previous to the period of excessive gain weight; third, determine which diet patterns are associated with the increased risk for obesity development.

## METHOD

This is a cohort study nested to a randomized field trial on breastfeeding and dietary practices (NCT00635453). A complete study of the original design was published previously.^18^ The intervention trial had institutional and educational nature and was conducted in primary care health centers that provided prenatal, infant, and other primary care services to low-income families in Porto Alegre, South Brazil. Briefly, employees (physicians, nurses, and administrative staff) of the health centers allocated in the intervention group participated in a lecture based on the “Ten Steps for Healthy Feeding for Brazilian Children from Birth to Two Years of Age” guideline^19^ which addressed strategies to best incorporate the dietary recommendations into the babies follow-ups. At the beginning of the study, Porto Alegre had 52 healthcare centers and 31 met the study criteria. Initially, 16 centers were selected by the principal investigator in a way that two centers would be included from each of the city’s eight geo-administrative districts. Aiming to maintain a balanced number of births by intervention and control groups, four additional centers from the original 31 were randomly drawn, which yielded nine intervention and eleven control centers. From April to December 2008, all pregnant women in the last trimester of prenatal care in the health centers were invited to participate in the study. During this recruitment, socioeconomic data, as well as the addresses and family characteristics were obtained. All births occurred between May 2008 and February 2009. After that, mothers were interviewed at home visits by trained interviewers when their children were aged six months, 12 months, three years, and six years. Data collected during home visits were verified through telephone callbacks to a 5% random sample of interviewed mothers. The analyses for this study were conducted between 2017 and 2018 and included children from both intervention and control groups.

The sample size of the umbrella study was calculated to detect a difference in the prevalence of exclusive breastfeeding at four months.^20^ A power of 90%, an α of 0.5, a design effect of 1.5, and a loss prediction of 20% were used to calculate the sample size, which came to 715 pregnant women. The study was approved by the Ethics in Research Committee of the Universidade Federal de Ciências da Saúde de Porto Alegre (UFCSPA) and the Ethics Committee of the Prefeitura Municipal de Porto Alegre, and the study was registered on the ClinicalTrials.gov website under the identification number NCT00635453. All mothers provided informed consent on behalf of their children.

Birth weight, length, and sex were collected from the children’s health records provided by maternities. At ages six months and 12 months, children were weighed during home visits without clothing on a portable digital scale (Techline^®^, São Paulo, Brazil), and length was measured using an infant stadiometer (SECA^®^, Hamburg, Germany). Children at three and six years were weighted at home or at the institution, barefoot and wearing light clothes on a digital scale (Techline^®^, São Paulo, Brazil and Líder^®^, São Paulo, Brazil, respectively) to the nearest 0.1 kg. Height was measured standing on a stadiometer (SECA^®^, Hamburg, Germany, and AlturaExata^®^, São Paulo, Brazil, respectively) to the nearest 0.1 cm. Age- and sex-specific body mass index-for-age (BMI-for-age) z-score, length/height-for-age z-score (HAZ-for-age), and cutoffs were determined by using the WHO Child Growth Standards (2006) and WHO Anthro software (www.who.int/childgrowth/software/en/).

Changes in weight gain measurements from birth to six years of age were analyzed as >0.67 BMI-for-age z-score variation, as it represents the difference between the percentile lines displayed on standard growth charts.^21^ Overweight and obesity were defined as a BMI z-score >1SD and >2SD for all ages, respectively.

Dietary intake information was estimated by a multiple-pass 24-hour dietary recall applied by nutritionists and undergraduate students with standardized training. Mothers or caregivers provided information about all foods and beverages consumed by their infants during the previous day. Details about food types, amounts, and preparation methods were recorded. Standard household measures (e.g., teaspoons, tablespoons, cups, and serving sizes) were used to help mothers report the amounts of food given to their children and standardize portion sizes. The research supervisor reviewed all the dietary recalls. The dietary data were estimated using the Dietwin^®^ software program (version 2008 professional Dietwin^®^), and the Brazilian Food Composition Table was preferentially used as a reference, followed by the United States Department of Agriculture Chemical Composition Tables. For commercial products, we manually added all nutritional compositions provided by the manufacturer to the program.

We assigned all food and beverage items to 15 food groups based on their similarity in nutrient content or culinary usage for dietary pattern analysis. To determine the dietary patterns, we applied principal components analysis. The factors obtained were linear combinations of the included variables, explaining as many variations in the original variables as possible. The 15 food groups were expressed in milliliters or grams. After that, orthogonal rotation (Varimax) was applied to simplify the data structure and clarify interpretations.^22^ The orthogonal analysis refers to the use of statistical methods in exploratory factor analysis (EFA) to identify distinct patterns within data sets, such as food groups. Finally, the number of factors selected was based on two strategies: eigen value >1 and the scree plot assessment. Food subgroups with a factor loading greater than 0.30 or less than 0.30 were considered to identify each pattern. Sample adequacy was checked using the Kaiser-Meyer-Olkin (KMO) test. The KMO can assume values between 0 and 1. Low values mean that the variables have too little in common to proceed with the analysis. Values below 0.5 are considered unacceptable. In the present study, we obtained a KMO=0.603.

As this study is derived from a randomized field trial, we verified potential differences between intervention and control groups concerning the incidence of excessive weight gain, and no differences were found from birth to six months of age (p=0.291), six months to 12 months (p=0.532), 12 months to three years (p=0.106), and three years to six years of age (p=0.204). Continuous variables were expressed as mean and standard deviation (normally distributed data) or median and interquartile range (non-normally distributed data) and percent frequency. The excessive weight gain in all ages was described using percentages and compared by logistic regressions using a Generalized Estimating Equation with a binomial probability distribution and logit link function. Odds ratios and 95% confidence intervals for the risk of overweight and obesity were examined by comparing the frequency with which children reached or did not reach specific BMI z-scores at three and six years of age.

Poisson regression analysis was used to assess the effect of each diet pattern in quartiles and excessive weight gain. The model was adjusted for the child’s group status (intervention or control), dietary patterns, exclusive breastfeeding until four months, birth weight (in grams), gestational weight gain, and smoking during pregnancy. Data were expressed using percentage, relative risk (RR), and 95% confidence intervals (95% CI). All statistical analyses were conducted using Statistical Package for the Social Sciences (SPSS) version 21.0 (IBM Statistics Inc, Chicago, IL, USA), and statistical significance was set at p<0.05.

## RESULTS

**Figure 1:**
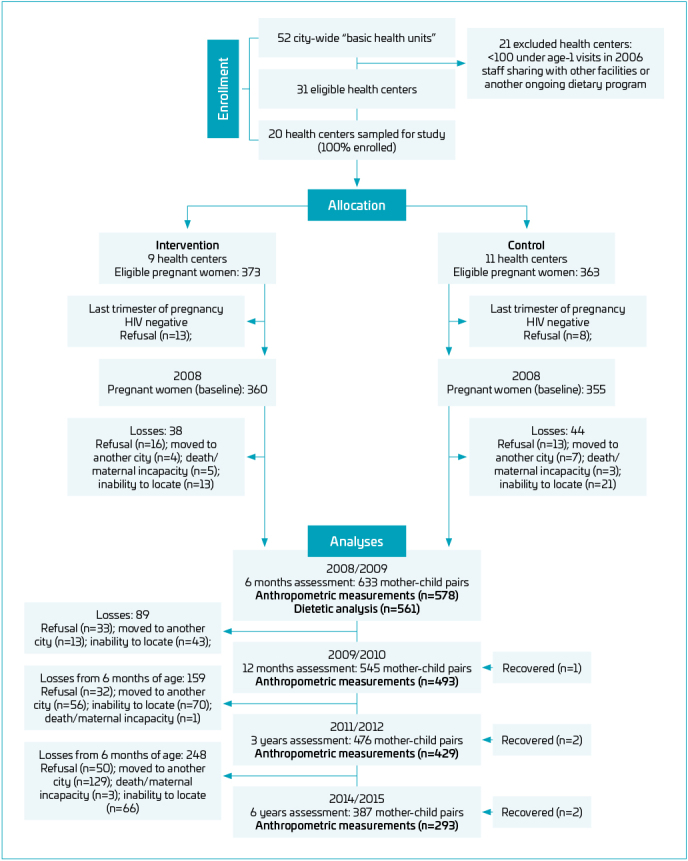
Flow diagram of the study conducted with pregnant women and later assessments at 6 and 12 months, and 3 and 6 years of their children.

At baseline, 715 pregnant women were recruited. About 633 children underwent the assessment at the age of six months, 545 at 12 months, 476 at three years, and 387 at six years. For this study, premature births and children with genetic and congenital diseases were excluded from the analyses (Figure 1). Complete anthropometric data were available for 568 infants at birth, 578 at age six months, 493 at 12 months, 429 at three years, and 293 at six years. The comparison of baseline data between the analytic sample and those who withdrew from the study revealed no statistically significant differences regarding maternal age, maternal education, maternal employment, maternal nutritional status, father’s education, father’s employment, family structure, number of people living in the house and monthly family income, as described previously.^23^

Demographic characteristics of children lost to follow-up were compared to those who remained at six years of age. No differences were found for maternal age at the child’s birth, maternal education level, and maternal employment (p>0.050). Of 715 women at baseline, 20.7% were under 20 years of age at the child’s birth, 30.8% had less than eight years of schooling, and 66.9% did not have paid employment. Family income was low for most families — 70.3% of those who reported had a monthly income less than three times the national minimum wage (approximately US$ 565.00/month). More than half of the children were male (52.6%), and 36.5% were non-white (data not shown in tables).

Excessive weight gain trajectories were analyzed at four age periods: from birth to six months (n=565); from six to 12 months (n=490); from 12 months to three years (n=392); and from three to six years (n=270). The critical period for excessive weight gain was observed from six to 12 months of age (46.5% BMI variation >0.67), where the percentage of children with rapid weight gain (z-score>0.67) was significantly higher than any other age time point (p<0.001). At other ages, the percentage of excessive weight gain ranged from 34.3% at birth to six months to 18.5% at three to six years of age. The percentage of overweight was 20.6% at birth, 28.9% at age six months, 47.8% at 12 months, 43.9% at three years, and 33.4% at six years (Table 1). Additionally, children in the BMI z-score >1 standard deviation (SD) at 12 months had three times more chance to have BMI z-score >2SD at three years (odds ratio — OR = 2.93; 95%CI 1.54–5.58) and seven times more chance to have BMI z-score >2SD at six years of age (OR = 7.57; 95%CI 3.92–14.60) (data not shown in tables).

**Table 1:** Anthropometric measurements and nutritional status in children at birth, 6 months, 12 months, 3 years, and 6 years of age.

		Birth		6 months		12 months		3 years		6 years	
		n	mean (SD)	n	mean (SD)	n	mean (SD)	n	mean (SD)	n	mean (SD)
Weight (kg)		611	3.28 (0.50)	616	8.00 (1.09)	527	10.10 (1.35)	460	15.79 (2.57)	316	24.05 (5.61)
Length/Height (cm)		606	49.04 (2.45)	617	67.10 (2.75)	529	74.77 (3.01)	456	96.71 (4.43)	316	119.07 (5.71)
		**n**	**%**	**n**	**%**	**n**	**%**	**n**	**%**	**n**	**%**
Length/Height-for age z-scores (HAZ)	<-2	42	7.4	22	3.8	16	3.2	17	3.9	4	1.2
BMI-for-age z-scores (BAZ)	<-2	16	2.8	15	2.6	1	0.2	4	0.9	3	1.0
	≥-2 <-1	41	7.2	60	10.4	20	4.1	15	3.5	25	8.4
	≥-1 ≤1	394	69.4	336	58.1	236	47.9	222	51.6	170	57.2
	>1 ≤2	102	18.0	119	20.6	150	30.4	109	25.3	45	15.2
	>2	15	2.6	48	8.3	86	17.4	80	18.6	54	18.2
**n/total (%)**											
BMI-for-age variation (>0.67)^a^	Birth to 06 months	194/565	(34.3)	-	-	-	-	-	-	-	-
	06 months to 12 months	-	-	**228/490**	**(46.5)**	-	-	-	-	-	-
	12 months to 03 years	-	-	-	-	106/392	(27.0)	-	-	-	-
	03 years to 06 years	-	-	-	-	-	-	50/270	(18.5)	-	-

SD: Standard Deviation; BMI: Body Mass Index; p<0.05; ^a^Generalized Estimation Equation.

Regarding dietary assessment at six months of age, 20 infants were excluded from the analysis because they were exclusively were on exclusive breastfeeding (n=18) or sick (n=2). Thus, dietary data were available for 561 infants. Five dietary patterns were identified. Three out of five were predominantly composed of ultra-processed foods. The first pattern showed positive loadings for cow milk, sugar, and/or baby cereal, with negative loadings for breastmilk, thus earning the label “baby bottle”. The second pattern exhibited positive loadings for soda and sweetened juice, labeled as “sweet beverages”. The third one showed positive loading for fresh juice and creamy dessert, called petit suisse, which labeled as “dessert”. Lastly, the remaining two patterns displayed positive loadings for a variety of whole foods, the first one with positive loadings for roots, tubers, meat, eggs, fruits, and vegetables, that were labeled as “table foods”, and the second one with positive loading for rice and pasta, beans, bread, and baby biscuits, that labeled “cultural baby food”. The factor loadings are presented in Table 2.

**Table 2:** Relative risk for excessive weight gain* according to the quartiles of dietary pattern (n=561)^†^.

Q	Univariate model^‡^		Multivariate model			
	RR (95%CI)	p-value	Model 1^§^ RR (95%CI)	p-value	Model 2^//^ RR (95%CI)	p-value
Bottle (cow milk. sugar and baby cereal flour) and lower or no breastmilk						
Q1	1		1		1	
Q2	0.88 (0.70–1.10)	0.289	0.90 (0.73–1.11)	0.363	0.90 (0.73–1.11)	0.347
Q3	1.30 (1.05–1.62)	0.015	1.37 (1.13–1.67)	≤ 0.01	1.43 (1.15–1.79)	≤0.001
Q4	1.34 (1.10–1.63)	0.003	1.43 (1.16–1.77)	≤ 0.01	1.59 (1.28–1.97)	≤0.001
Roots and tubers. Meat and eggs. Fruits and vegetables						
Q1	1		1		1	
Q2	0.97 (0.73–1.29)	0.869	0.98 (0.73–1.32)	0.926	0.99 (0.74–1.32)	0.970
Q3	0.79 (0.58–1.08)	0.143	0.85 (0.62–1.17)	0.331	0.82 (0.59–1.13)	0.234
Q4	1.00 (0.84–1.19)	0.925	1.08 (0.91–1.28)	0.362	0.99 (0.74–1.32)	0.970
Rice and pasta. Beans. Bread. Baby biscuits						
Q1	1		1		1	
Q2	0.97 (0.80–1.17)	0.787	1.12 (0.88–1.42)	0.331	1.07 (0.89–1.29)	0.430
Q3	1.05 (0.83–1.33)	0.667	1.16 (0.93–1.46)	0.184	1.18 (0.93–1.49)	0.155
Q4	0.90 (0.76–1.06)	0.213	0.97 (0.81–1.16)	0.775	1.15 (0.90–1.46)	0.249
Sweet beverages (soda. Sweetened juice)						
Q1	1		1		1	
Q2	1.16 (0.99–1.37)	0.064	1.20 (1.04–1.38)	≤0.01	1.19 (1.02–1.38)	0.024
Q3	1.04 (0.93–1.15)	0.438	1.07 (0.91–1.26)	0.396	1.06 (0.88–1.27)	0.534
Q4	1.30 (1.05–1.61)	0.016	1.29 (0.99–1.69)	0.052	1.31 (0.98–1.74)	0.060
Fresh juice and creamy dessert						
Q1	1		1		1	
Q2	1.34 (1.05–1.70)	0.016	1.33 (1.05–1.69)	≤ 0.01	1.39 (1.08–1.33)	≤0.001
Q3	1.18 (0.97–1.44)	0.097	1.14 (0.92–1.40)	0.208	1.19 (0.92–1.53)	0.178
Q4	1.15 (0.96–1.39)	0.114	1.06 (0.87–1.29)	0.534	1.05 (0.83–1.33)	0.652

RR. relative risk; CI: confidence interval.

*ΔBMI Z-score >0.67

^†^Poisson regression analysis

^‡^Univariable model: â-coefficients for a separate model for each listed variable.

^§^Multivariate model 1: adjusted for child’s group status (intervention or control) and dietary patterns;

^//^Multivariate model 2: adjusted for child’s group status (intervention or control), dietary patterns, exclusive breastfeeding until 4 months, birth weight (in grams), gestational weight gain, smoking on pregnancy.

In multivariate analysis, excessive weight gain was associated with dietary patterns that included sugar and ultra-processed foods, such as the third and fourth quartiles of baby bottle pattern and the second quartile of dessert, and sweet beverages pattern. These findings persisted after adjustment for the other dietary patterns, exclusive breastfeeding until four months of age, birth weight (in grams), gestational weight gain, and smoking during pregnancy (quartiles of baby bottle pattern: relative risk — RR 1.43; 95%CI 1.15–1.79; p≤0.001 and RR 1.59; 95%CI 1.28–1.97, p≤0.001; respectively; quartile of fresh juice and creamy dessert and sweet beverage patterns: RR 1.39; 95%CI 1.08–1.33; p≤0.001 and RR 1.19; 95%CI 1.02–1.38; p=0.024, respectively) (Table 3).

**Table 3:** Factor loadings extracted by principal component analysis among children at 6 months of age (n=561).

	Dietary patterns				
	Bottle	Roots and tubers. Meat and eggs. Fruits and vegetables	Rice and pasta. Beans. Bread. Baby biscuits	Sweet beverages	Fresh juice and creamy dessert
Breast milk	-0.756	-0.131	-0.123	0.026	-0.202
Cow milk	0.842	-0.017	0.086	0.049	-0.016
Rice and pasta	0.008	0.087	0.698	0.224	0.194
Roots and tubers	0.157	0.565	0.107	0.107	-0.392
Meat and eggs	-0.044	0.608	0.098	0.406	0.316
Beans	0.079	-0.113	0.735	-0.062	0.074
Fruits	-0.051	0.517	0.059	-0.089	0.023
Vegetables	0.018	0.728	-0.126	-0.024	0.056
Fresh juice	0.032	0.304	0.017	-0.483	0.491
Bread*	-0.047	-0.306	0.376	0.334	0.177
Baby biscuits^†^	0.124	0.227	0.574	-0.154	-0.297
Sweet beverages^‡^	0.099	0.077	0.013	0.743	0.021
Creamy dessert	0.148	0.005	0.113	0.075	0.607
Sugar	0.641	0.008	-0.012	0.051	0.070
Baby cereal flour	0.768	-0.078	0.001	0.007	-0.079

*mass-produced packaged bread and buns; french bread; salt biscuits and crackers

^†^baby biscuits and cookies

^‡^soda; sweetened juice.

## DISCUSSION

The critical period for excessive weight gain was observed between six and 12 months, since approximately 50% of the infants from six months to 12 months of age presented rapid weight gain (>0.67 BMI z-score). The analysis of food consumed by children at six months of life revealed three dietary patterns associated with excessive gain at 12 months of age, labeled as “baby bottle”, “sweet beverages” and “dessert”.

Despite the high frequency of children with excessive weight gain in the first six months of life, approximately 50% of the infants from six to 12 months of age presented rapid weight gain (>0.67 BMI z-score) in our study, which is a robust predictor of childhood overweight and obesity. When excessive weight gain occurs early in life, the bowel permeability and metabolic malleability in early childhood are damaged,^24^ modifying endocrine interactions and changing the infant’s state of inflammatory and oxidative stress.^25^ Such biological changes in infancy, including insulin resistance and fat accumulation in a critical growth period, could provide a metabolic profile susceptible to obesity development, explaining the higher risk for later overweight and obesity.^26^

Based on the anthropometric results described before, we investigated the dietary patterns at age six months and their effects on the rapid weight gain in the second semester of life. Three of the five dietary patterns found in our child sample were associated with infants’ excessive weight gain, all containing at least one ultra-processed food, according to the NOVA classification system.^27^ The first was the baby bottle with cow milk, baby cereal flour, and sugar, the second was fresh juice and creamy dessert, and the third was soda and sweetened juice (called sweet beverages pattern).

Some hypotheses can be driven, considering each specific pattern and their impact on the development of excess weight gain. The bottle-feeding pattern, characterized by cow milk with sugar and baby cereal flour, was the strongest one compared to the others. This pattern facilitates excessive energy intake, and the high protein content associated with the increased insulin-releasing amino acids plasmatic concentration, stimulating the secretion of insulin and insulin-like growth factor I (IGF-I), enhancing weight gain and body fat deposition.^28^ Besides, the use of the baby bottle itself may contribute to the infant’s poor self-regulation appetite. The practice of baby bottle feeding can be a more passive process driven by caregivers, less responsive to infants’ internal cues of hunger and satiety.^29^ Although a large body of evidence illustrates that bottle-feeding itself is a significant predictor of excessive weight gain, the content of the baby bottle in developed countries is usually infant formula. In the present study, the bottles usually contained cow’s milk added with sugar and/or baby cereal flour, which is a dietary typical pattern in Brazil,^30^ and which may be more pronounced in low-income groups in which the culture of that heavy baby is a healthier baby, leading to obesogenic practices.^31^

The second and third patterns (sweet beverages; fresh juice and creamy dessert) were associated with excessive weight gain but only for children consuming the 2^nd^ quartile and not the highest quartiles. This is a challenging result that leads to two possible explanations. First, the association found for both patterns with excessive weight gain has some physiological hypothesis: a weak compensation of energy intake by liquids, lack of fiber, rapid absorption of fructose and glucose facilitates extra energy intake, promotes high glycemic load, increase insulin production, and causes rapid fructose absorption by the liver.^32^ The second explanation for the lack of association between excessive weight gain and the highest quartiles may be supported by the evidence that excessive intake of sweet beverages promotes a reduction in the child’s appetite for other energy and nutrient-dense foods, nullifying the effect on extra energy consumption.^33,34^

In addition, all three of these patterns have in common a high concentration of energy and added sugar, making them more palatable and very easy to eat without much effort to chew or swallow. These data are concerning because babies are born with innate preferences for sugar and biologically will prioritize meeting their primal hunger needs, which fundamentally means preferring calories over other nutritional needs. The inappropriate introduction of solid foods and sweet beverages also raises the infant’s risk for later obesity and may discourage the acceptance of other bitter or sour foods, such as fruits and vegetables.^35^ Considering this, foods added with sugars are non-nutritive and non-recommended in children under two years of age.^36^

Therefore, these dietary patterns at six months of age could be the promoters of a higher intake of energy and, consequently, excessive weight gain up to 12 months observed in this study. These findings are consistent with data from a recent study with a representative sample of US infants which showed that infants with dietary patterns rich in ultra-processed foods were more likely to present rapid weight gain and overweight/obesity risk at six and 12 months of age.^13^ The establishment of excessive adipose mass implies a new metabolic pathway which will work towards energetic homeostasis (regulated by central nervous and endocrine systems and fat stores) to reset the body weight in the increased value. Thus, at an early age such as 12 months, this new metabolic pattern, settled by the overweight condition, will generate a cascade of signals to increase appetite and decrease satiety, resulting in a continuous higher energy intake.^37,38^

Finally, we would like to highlight the strengths of this study. It was carried out from birth to six years of age, which made it possible to identify the period of greatest risk for excessive weight. Moreover, the dietary assessment carried out at six months of age allowed us to reveal an early causality of childhood obesity. On the other hand, some limitations of our study should be also pointed out. First, there were losses during follow-up. However, there were no significant differences between the baseline characteristics of children who remained in the study and those lost to follow-up. Second, cautious generalization is required, since most of our sample had low family income and may limit the applicability of our findings to more privileged populations. Third, a single 24-hour recall might be limited in capturing the usual intake. However, considering the period of 6–9 months in which there are not many variations in food intake, we do not think that this aspect interfered with the result. Fourth, we did not include children’s sleep patterns and physical activities in the confounding variables; however, due to the early age of those included in the dietary analysis, we believe that there are no differences among children in this period. Finally, an important point to consider is that this cohort was initially designed as a randomized field trial, which means that an intervention was carried out to health professionals who assisted the mother-child pairs participating in the study. Significant results were observed in improving food quality, reducing added sugar and energy intake consumption, and reducing children’s adiposity measures.

In conclusion, the period from six to 12 months of age was critical for excessive weight gain during the first six years of the life of children in a low-income community in Brazil. Dietary patterns at six months of age, associated with the subsequent period of excessive weight gain, were mainly represented by bottle feeding, fruit juice, and sweet beverages. Thus, the results highlight the importance of early intervention in childhood obesity prevention, since it is an easier path than its treatment. Moreover, the existing national guidelines and public policies can be better implemented and transformed into effective actions in all healthcare settings, with special attention to the primary care level. Health professionals should focus on warning parents about the health risks of early introduction of sugar and ultra-processed foods, along with routine guidance on healthy complementary foods.

## Data Availability

The database that originated the article is available with the corresponding author.
